# Wnt Signaling in Gynecologic Malignancies

**DOI:** 10.3390/ijms21124272

**Published:** 2020-06-16

**Authors:** Alexandra McMellen, Elizabeth R. Woodruff, Bradley R. Corr, Benjamin G. Bitler, Marisa R. Moroney

**Affiliations:** 1 Department of OB/GYN, Division of Reproductive Sciences, The University of Colorado, Aurora, CO 80045, USA; alexandra.mcmellen@cuanschutz.edu (A.M.); elizabeth.r.woodruff@cuanschutz.edu (E.R.W.); benjamin.bitler@cuanschutz.edu (B.G.B.); 2 Department of OB/GYN, Division of Gynecologic Oncology, The University of Colorado, Aurora, CO 80045, USA; bradley.corr@cuanschutz.edu; 3 University of Colorado Comprehensive Cancer Center, Aurora, CO 80045, USA

**Keywords:** gynecologic malignancies, Wnt signaling, ovarian cancer, endometrial cancer, cervical cancer

## Abstract

Gynecologic malignancies, including ovarian cancer, endometrial cancer, and cervical cancer, affect hundreds of thousands of women worldwide every year. Wnt signaling, specifically Wnt/β-catenin signaling, has been found to play an essential role in many oncogenic processes in gynecologic malignancies, including tumorigenesis, metastasis, recurrence, and chemotherapy resistance. As such, the Wnt/β-catenin signaling pathway has the potential to be a target for effective treatment, improving patient outcomes. In this review, we discuss the evidence supporting the importance of the Wnt signaling pathways in the development, progression, and treatment of gynecologic malignancies.

## 1. Introduction

Wnt signaling is one of the most studied signaling pathways and plays a critical role in multiple biological processes, including cell differentiation, proliferation, survival, and migration. There is significant evidence that Wnt signaling also plays a pivotal role in tumorigenesis and other oncogenic processes [1,2,3]. The Wnt signaling pathway is tightly regulated through both stimulatory and inhibitory mechanisms. When these regulatory mechanisms are lost or altered through mutations, Wnt signaling is aberrantly activated, resulting in deregulated cellular processes [2,4]. The hyperactivation of Wnt signaling has been implicated in tumorigenesis and cancer progression in several different cancer types, and Wnt target genes have been associated with tumor proliferation, metastasis, epithelial-to-mesenchymal transition (EMT), recurrence, chemoresistance, and anti-tumor immune regulation [2,3,5,6]. 

More recently, there is growing research evaluating the role of Wnt signaling in gynecologic malignancies. Specifically, Wnt signaling promotes metastasis and therapy resistance in ovarian cancer, plays a crucial role in tumorigenesis and recurrence in endometrial cancer, and participates in human papillomavirus (HPV) -related tumorigenesis and metastasis in cervical cancer [7,8,9,10,11,12,13,14,15,16,17,18,19,20,21,22,23,24,25]. For all of these gynecologic malignancies, Wnt signaling is also being evaluated as a possible therapeutic target [26,27,28,29,30,31,32,33]. In this review, we will discuss the Wnt signaling pathway as it is related to the development, progression, and treatment of gynecologic malignancies. 

## 2. Wnt Signaling

There are three well-defined Wnt signaling pathways separated into two categories: canonical (β-catenin-dependent) and non-canonical (β-catenin-independent). The canonical pathway is a fundamental growth control pathway known to have important roles in many fields, including cancer. The non-canonical pathways mediate cell polarity and regulate levels of intracellular calcium [34].

There are 19 mammalian protein-encoding Wnt genes, many with unique functions. Wnt proteins undergo several post-translational modifications, including palmitoleoylation, which is accomplished by the palmitoyl transferase, Porcupine (PORCN). These modifications are critical for Wnt extracellular signaling, rendering the proteins hydrophobic so that they may be secreted from the cell. The transmembrane protein Wntless then traffics the Wnt proteins to the plasma membrane where they are secreted to signal targeted cells nearby. Wnt signaling then continues at the target cell membrane of the targeted cell where Wnt proteins bind to the Frizzled (FZD) transmembrane receptor and recruit the Dishevelled protein (DVL). There are 10 FZD receptors and at least 5 co-receptors (LRP5/6, ROR1/2, RyK). The specific FZD and co-receptors engaged by the Wnt ligand tailor the initiation of intracellular signaling and determine which Wnt signaling pathway, canonical or non-canonical, proceeds [3,34,35]. 

### 2.1. Non-Canonical Pathways—β-Catenin Independent

The non-canonical Wnt signaling pathways are introduced here. However, as the majority of Wnt signaling aberrations in gynecologic malignancies are in the canonical pathway, the majority of this review will focus on the canonical Wnt signaling pathway. 

#### 2.1.1. Planar Cell Polarity

There are six main components of the Planar Cell Polarity (PCP) pathway known as the core module. These components consist of three transmembrane proteins (FZD, Vangl, and Celser) and three cytoplasmic proteins (DVL, Prickle (Pk), and Diversin). These components ultimately activate the small Rho GTPase effector molecules, c-Jun N-terminal kinase (JNK) and Nemo-like kinase (NLK). The PCP Wnt pathway components are arranged asymmetrically within the cell in order to influence cell polarity [36]. Determination of cell polarity during development is vital for proper cell organization and tissue function. Defects in PCP signaling have been linked with some developmental diseases including polycystic kidney disease, heart defects, and deafness [36,37,38,39].

#### 2.1.2. Calcium-Dependent Wnt Pathway

The calcium-dependent Wnt pathway controls gene expression by modulating intracellular levels of calcium [36]. This intracellular pathway is also initiated by Wnt ligand proteins, most commonly WNT5A, which has been implicated as a tumor suppressor in multiple cancer types [40,41,42,43]. In mice, the binding of WNT5A ligand to FZD and activation of the co-receptor ROR2 tyrosine kinase inhibit canonical Wnt signaling [44]. DVL and G proteins activate phospholipase C leading to increased diacylglycerol (DAG), inositol 1,4,5-triphosphate (IP3), and intracellular calcium [36]. The increase in these signaling molecules subsequently leads to the activation of calcium calmodulin-dependent protein kinase II (CaMKII) and protein kinase C (PKC). CaMKII and PKC consequently activate the transcription factors NFκB and CREB [36]. 

### 2.2. Canonical Pathway—β-Catenin Dependent

At the initiation of the intracellular canonical Wnt signaling pathway (also called the Wnt/β-catenin signaling pathway), Wnt proteins bind to FZD as part of a larger receptor complex at the cell membrane. LGR4, LGR5, and LGR6 are members of the 7-transmembrane receptor family and are able to bind R-spondins (RSPO) with high affinity in order to enhance the Wnt signal when there is a low dose of Wnt ligand [34]. The receptor complex also includes lipoprotein receptor-related protein (LRP) receptors. Wnt protein-receptor interactions induce the sequestration of DVL proteins, leading to the disassembly of the β-catenin degradation complex and subsequent accumulation of cytosolic β-catenin. The β-catenin proteins then translocate to the nucleus via BCL9 and Pygo1 [45] where β-catenin interacts with the T-cell factor (TCF) and lymphoid enhancer factor (LEF) transcriptional activators resulting in upregulation of TCF/LEF target genes. In the absence of Wnt protein-mediated signaling, intracellular β-catenin levels are regulated by the degradation complex. The β-catenin protein is trapped by the degradation complex, which is composed of Axin, APC, GSK3β, and CK1α. Within the degradation complex, GSK3β and CK1α phosphorylate β-catenin, promoting its ubiquitination and subsequent proteasomal degradation [3,34,35]. 

The Wnt/β-catenin signaling pathway has multiple identified regulatory mechanisms in addition to phosphorylation and ubiquitination of β-catenin by the degradation complex, as previously described [35]. Negative regulatory mechanisms include the protein Notum, which removes palmitoleate from Wnt proteins, preventing their extracellular secretion; Dickkopf (DKK) proteins that competitively bind to LRP5/6 receptors blocking the initiation of Wnt protein-mediated signaling; secreted Frizzled related proteins (sFRPs), which bind to FZD receptors also blocking the initiation of Wnt protein-mediated signaling; Wnt Inhibitory Factor (WIF), which inhibits signaling by binding directly to Wnt proteins [36]; the transmembrane molecules ZNRF3 and RNF43 which have E3 ubiquitin ligase activity and act on FZD molecules leading to their turn over [34].

Numerous mechanisms of Wnt/β-catenin hyperactivation have been described. Overexpression of Wnt ligands results in increased Wnt signaling [46]. Intracellularly, mutations within the destruction complex (e.g., APC) prevent the phosphorylation and subsequent ubiquitination of β-catenin [2]. Aberrant activation of PI3K (e.g., PIK3CA mutations), which is detected in many ovarian cancers, can result in inhibitory phosphorylation of GSK3β, rendering it unable to phosphorylate β-catenin and thus ultimately preventing β-catenin degradation [35]. Hotspot mutations in Exon 3 of the *CTNNB1* gene (encodes for the β-catenin protein) alter the N-terminus of β-catenin and prevent its phosphorylation and degradation by the destruction complex. Concerning gynecologic malignancies, *CTNNB1* mutations are detected in uterine endometrial, ovarian endometrioid, and ovarian clear cell carcinomas [7,47,48]. Hyperactivation of Wnt/β-catenin signaling through these mechanisms are implicated in tumorigenesis, tumor progression, recurrence, and chemoresistance in several cancers, including gynecologic malignancies [7,8,26,49,50].

## 3. Ovarian Cancer and Wnt Signaling

Epithelial ovarian cancer (EOC) is the 5th leading cause of cancer-related death in women in the United States [51]. High grade serous ovarian cancer (HGSOC) is the most common histotype of EOC, accounting for over 70% of cases, and it mainly arises from fallopian tube epithelial cells [52]. The majority of HGSOC cases present at a later stage (III or IV) and have a poor prognosis (5-year survival <50%) due to difficulty in diagnosis, high recurrence rates and development of therapy resistance [53,54,55]. Once diagnosed, patients with HGSOC are frequently treated with cytoreductive surgery and platinum-based adjuvant chemotherapy. Approximately 80% of patients ultimately develop recurrent disease and eventual platinum chemotherapy resistance, limiting the options for and success of future treatment lines [53,55]. Improving our understanding of EOC tumorigenesis, metastasis, disease progression, and therapy resistance will allow for advancements in early diagnosis and therapeutics and ultimately, an improvement in patient outcomes. 

Wnt/β-catenin signaling plays a role in HGSOC tumorigenesis, metastasis, and therapy resistance. According to The Cancer Genome Atlas (TCGA), while mutations in the pathway are rare, gene amplification or deletions of Wnt signaling components (148 genes, excluding *TP53* and *MYC*, [56]) are detected in approximately 88% of HGSOC tumors (Table 1) [57]. For instance, DVL3, and LRP6 are amplified in 27% and 10% of cases, respectively. This suggests that the Wnt/β-catenin signaling pathway can be hyperactivated in HGSOC through the amplification of pathway activators or deletions of pathway suppressors. 

### 3.1. Tumorigenesis

Both canonical and non-canonical Wnt signaling can promote ovarian cancer cell survival in specific contexts. Wnt/β-catenin signaling promotes survival in HGSOC cells and its inhibition leads to impaired proliferation and migration [27]. For example, the Wnt ligand WNT10A activates canonical Wnt signaling in ovarian cancer cell lines leading to increased viability and cell migration [58]. Additionally, the poly(ADP) ribose polymerase (PARP) Tankyrase (TNKS) activates canonical Wnt signaling in ovarian cancer independent of Wnt ligands through destabilization of the destruction complex [49]. In this context, TNKS-activated Wnt/β-catenin signaling contributes to colony formation, migration, invasion, and tumorigenic potential of ovarian cancer cell lines, as well as the promotion of aerobic glycolysis, which is often observed in malignant cells [49]. Similarly independent of Wnt ligands, RSPO1 promotes ovarian cancer cell survival and migration through upstream activation of the Wnt/β-catenin signaling pathway [59]. When either TNKS or RSPO1 are inhibited, Wnt/β-catenin signaling activity decreases and ovarian cancer cells undergo apoptosis [49,59]. 

The regulation of canonical and non-canonical Wnt signaling is complex and dysregulation resulting in hyperactivation or loss of different components of the signaling pathways can ultimately lead to tumorigenesis or disease progression. For example, activation of non-canonical Wnt signaling by overexpression of WNT5A (non-canonical PCP Wnt protein) in ascites fluid promotes metastatic stem-cell like behavior in HGSOC cells and results in worse survival [60]. Our group demonstrated that WNT5A was significantly underexpressed in primary human EOC compared to normal ovarian surface and fallopian tube (FT) epithelial tissue and that loss of WNT5A correlates with worse survival [42]. In EOC cell lines and tumors, WNT5A overexpression induces cellular senescence, a tumor-suppressive pathway, and antagonizes the β-catenin-dependent transcriptional activity. The regulation of canonical and non-canonical Wnt signaling is complex and dysregulation resulting in hyperactivation or loss of different components of the signaling pathways can ultimately lead to tumorigenesis or disease progression [42,60].

### 3.2. Metastasis

As previously mentioned, HGSOC is usually diagnosed at a late stage in which the tumor has metastasized to multiple sites. Most HGSOC arise via a non-classical metastatic process, which starts in a *TP53*-mutation lesion in the fimbriated end of the FT, then causes cancer cells to accumulate there and subsequently exfoliate, resist anoikis, and colonize the peritoneal cavity. This metastatic process appears to be mostly independent of the vasculature and lymphatics [55]. 

The epithelial to mesenchymal transition (EMT) is the process by which epithelial cells transform into a more motile, invasive mesenchymal phenotype. It conveys resistance to anoikis in cancer cells, thus promoting cancer cell migration and peritoneal metastasis in HGSOC [9]. Wnt/β-catenin signaling plays a crucial role in EMT and metastasis in many cancers, including ovarian [10,11]. In HGSOC cell lines, IL-8 promotes cell migration by activating Wnt/β-catenin signaling-mediated EMT [10]. Conversely, downregulation of Wnt/β-catenin signaling by Cyclin G2 results in inhibition of EMT, ultimately inhibiting cell proliferation, migration, and invasion [61].

Peritoneal metastasis of HGSOC is also promoted by WNT5A, which is abundant in ascites [60,62]. As previously mentioned, WNT5A activity (non-canonical PCP signaling) promotes the metastatic stem-cell like behavior of HGSOC cells and confers a poor prognosis. Similarly, WNT5A in the ascites of HGSOC induces the formation of metastatic peritoneal implants by promoting ovarian cancer cell adhesion to the peritoneum, as well as ovarian cancer cell migration and invasion. Host peritoneal and adipose tissue secrete the WTN5A protein and loss of the host *WNT5A* gene results in significantly reduced peritoneal metastasis [62]. Expression of various Wnt/β-catenin signaling pathway molecules—not just WNT5A—are associated with disease outcomes in metastatic HGSOC. The expression of these Wnt molecules is dependent on anatomic/metastatic site, highlighting the importance of the tumor microenvironment (TME) and indicating that Wnt signaling activity in HGSOC varies depending on this TME [63]. 

### 3.3. Therapy Resistance

Wnt/β-catenin signaling is involved in HGSOC chemotherapy resistance [8,12,13]. Leucine-rich-repeat containing G protein-coupled receptor 6 (LGR6, a known activator of Wnt/β-catenin signaling) is upregulated in HGSOC and associated with poor chemotherapeutic response. Consistently, downregulation of LGR6 in loss-of-function assays attenuates the chemotherapy resistance by decreasing Wnt/β-catenin signaling activity [12]. Inhibition of β-catenin signaling using PRI-724 (an inhibitor of β-catenin interactions with its co-activator, CREB Binding Protein (CBP)) is sufficient to resensitize cells to cisplatin chemotherapy [13]. Also, treatment with sFRP4 (a known Wnt antagonist) alone and in combination with chemotherapies conveys chemotherapy-sensitization and improves the efficacy of chemotherapies [8]. These studies highlight that inhibition of the Wnt/β-catenin signaling pathway may serve to overcome chemotherapy-resistant ovarian cancer. 

Another essential therapy in the management of HGSOC is PARP inhibitors (PARPi), which are approved for upfront maintenance therapy in all advanced cases [64,65,66,67,68]. However, patients commonly experience disease recurrence and eventually develop PARPi-resistant disease [69]. Several mechanisms driving PARPi resistance have been identified, including epigenetic modifications, BRCA reversion mutations, kinase activation, and Wnt/β-catenin signaling [26,69,70,71]. Our group and an independent group recently published that Wnt/β-catenin signaling hyperactivation can promote PARPi resistance [26,72]. Fukumoto et al. observed that methylation of FZD10 mRNA promotes β-catenin activity and PARPi resistance in BRCA-deficient HGSOC cells. We demonstrated that hyperactivation of the canonical pathway via WNT3A overexpression was sufficient to promote PARPi resistance and increase DNA damage repair. Both studies observed that treatment with a Wnt inhibitor (Pyrvinium Pamoate and XAV939, respectively) was able to resensitize HGSOC cells to PARPi and lead to reduced tumor size in vivo [26,72], indicating that the Wnt/β-catenin signaling pathway is a potential target for overcoming therapy resistance in HGSOC.

### 3.4. Immune Landscape

Tumor immune response plays a significant role in patient outcomes and can affect possible treatment strategies. Immunologically “hot” tumors refer to tumors that have immune cell infiltration, while “cold” tumors lack this immune response [73]. Specifically, increased T- and B-cell tumor infiltration conveys a better prognosis for patients with HGSOC [74,75]. In HGSOC tumors, increased Wnt/β-catenin signaling inversely correlates with an activated T-cell signature [76], suggesting Wnt/β-catenin signaling contributes to conveying a “cold” tumor immune microenvironment. Using a syngeneic immune-competent mouse model of HGSOC, Goldsberry et al. confirmed the negative correlation between Wnt signaling and T-cell infiltration [28]. Treatment with a PORCN inhibitor (CGX1321) decreased Wnt ligand secretion and, in turn, lead to increased T-cell, macrophage, and dendritic cell activity. This enhanced immune response was accompanied by decreased tumor burden and improved survival, suggesting that targeting Wnt signaling may lead to increased immune cell infiltration and heightened anti-tumor immunity [28]. In mouse models, Doo et al. demonstrated that EOC tumors treated with the PORCN inhibitor WNT974 had increased CD8+ T cells and enhanced functioning of infiltrating CD4+ and CD8+ T cells, indicating that inhibition of Wnt signaling with WNT974 has immunomodulatory effects in the tumor microenvironment of EOC [29].

Immune checkpoint blockade (ICB) strategies (e.g., anti-PD-L1) have conveyed limited benefit in patients with HGSOC compared to those with melanoma or lung cancer, but Wnt/β-catenin signaling may serve as a targetable pathway to improve response to ICB. For instance, in triple-negative breast cancer, Wnt/β-catenin signaling directly promotes the expression of *CD274* (PD-L1) [77]. While further investigation in ovarian cancer is needed, combined Wnt inhibition with ICB could be effective in HGSOC tumors. 

Beyond ICB, recent evidence suggests that the Wnt inhibitor DKK1 may serve as an immunotherapeutic target in ovarian cancer [78]. Overexpression of DKK1 altered the immune microenvironment of ovarian cancer, leading to decreased CD8+ T cells and natural killer cells and a reduction of interferon-gamma (IFNy) expression on activated CD8+ T cells. These reports provide a rationale for further investigation into the intersection of Wnt/β-catenin signaling and anti-tumor immune regulation.

### 3.5. Other Ovarian Cancer Histotypes

While mutations in the Wnt/β-catenin signaling pathway are rare in HGSOC, they have been observed in other histotypes of ovarian cancer, namely endometrioid and mucinous carcinomas [79]. In mucinous ovarian cancer, aberrant activation of the Wnt pathway induces chemoresistance [80]. Aberrant Wnt/β-catenin signaling is present in up to 40% of endometrioid ovarian carcinoma, most frequently due to *CTNNB1* mutations [81]. A *CTNNB1* mutational analysis of 149 ovarian cancer samples detected 16% of endometrioid tumors harbored activating *CTNNB1* mutations [82]. The *CTNNB1* mutations, often called hotspot mutations, are focused around serine and threonine residues in exon 3, which encode known sites on β-catenin that are phosphorylated by GSK-3β. The most commonly mutated residues are Ser33 and Ser37, in which serine is changed to cystine, phenylalanine or tyrosine [14,82,83,84,85]. Thus, they prevent β-catenin degradation by the destruction complex [84,85]. Hyperactivation of the Wnt/β-catenin signaling pathway via other genetic mutations, including *MYC, APC*, and *CREBBP*, promote cell malignant transformation in ovarian endometrioid carcinoma [86]. Dapper1 Antagonist of Catenin-1 (DACT1), an inhibitor of β-catenin, is underexpressed in EOC cell lines and tissue samples [87], and overexpressing DACT1 in a mucinous ovarian cancer cell line leads to smaller tumors in vivo, as well as significantly lower levels of critical mediators of the Wnt pathway, including DVL2, β-catenin, and phosphorylated GSK-3β [87]. All of this taken together demonstrates that targeting Wnt/β-catenin signaling may serve as a promising treatment strategy for EOC, regardless of histotype. 

## 4. Endometrial Cancer

Endometrial cancer (EC) is the most common gynecologic malignancy in the United States and is one of the only cancers with an increasing incidence and mortality [51,88,89]. Currently, histopathologic features of EC tumors (histologic subtype and grade, disease stage, myometrial invasion, lymphovascular invasion (LVSI)) are used for risk-stratification and management decisions. However, there is a growing body of research evaluating the molecular make-up of EC and postulating that molecular classification of EC could improve risk-stratification, prognostication and treatment, ultimately improving clinical outcomes for EC patients [14,90,91]. The Wnt/β-catenin signaling pathway is of particular importance in the classification and risk-stratification of EC with approximately 65% of EC tumors containing an alteration within the Wnt/β-catenin signaling pathway (Table 2).

### 4.1. CTNNB1 as a Molecular Marker

One of the first and most comprehensive studies to molecularly classify EC was TCGA, which evaluated 373 cases of EC using whole-genome sequencing, exome sequencing, microsatellite instability assays, copy-number analyses, and DNA methylation testing [83]. TCGA identified four distinct genomic subgroups among EC that had significantly different survival outcomes and recurrence rates: polymerase epsilon (POLE) ultramutated, microsatellite instability (MSI) hypermutated, copy-number low (CNL), and copy-number high (CNH). The CNL subgroup is classified by frequent *CTNNB1* exon 3 mutations, as well as microsatellite stability and overall low mutation rates. Following TCGA, multiple studies independently reproduced the molecular classification of EC into the same four distinct subgroups with the same clinical outcomes, emphasizing the prognostic implications of this molecular classification system [14,91,92,93,94].

Exon 3 mutation in the *CTNNB1* gene occur in approximately 20–25% of endometrioid EC [83,84,95,96,97]. As demonstrated by both TCGA and Liu et al., most *CTNNB1*-mutant EC have a low overall genomic mutational rate, indicating that the *CTNNB1* mutations have autonomous oncogenic relevance [14,83]. Among endometrioid EC, *CTNNB1* mutations have been associated with low-risk histopathologic features, including low-grade histology, lack of lymph node metastasis, absence of LVSI and lower rates of deep myometrial invasion [15,94]. *CTNNB1* mutations are also associated with younger age at diagnosis, which is also considered a low-risk factor in current risk-stratification [14,15,98].

Despite the association with histopathologic and clinical features considered to be low-risk in the current EC risk-stratification system, *CTNNB1*-mutant EC has poorer clinical outcomes [7,14,15,98]. Kurnit et al. demonstrated that *CTNNB1* mutations were associated with decreased recurrence-free survival in low grade, early-stage EC [15]. Our group evaluated low-risk EC in a case-control study comparing recurrent Grade 1 Stage I EC to matched non-recurrent controls and found that *CTNNB1* mutations occurred at significantly higher rates in the recurrent EC [7]. Liu et al. have similarly demonstrated that *CTNNB1*-mutant low-grade EC exhibits upregulation of the Wnt/β-catenin pathway and poorer overall survival [14]. The increased Wnt/β-catenin signaling activity in EC with *CTNNB1* mutations was also demonstrated in a proteogenomic analysis of 95 EC by Yongchao et al. [84]. Based on these data, *CTNNB1* mutations need to be further evaluated as a marker for risk-stratification among low-risk EC. 

As previously described, *CTNNB1* mutations result in the accumulation of the β-catenin protein, and subsequent translocation into the nucleus and increased transcriptional activity [84,85]. Ιmmunohistochemistry (IHC) analyses have shown that nuclear expression of β-catenin is significantly correlated with *CTNNB1* mutations and can distinguish *CTNNB1*-mutant EC from wildtype with a sensitivity of 91% and 85% and a specificity of 89% and 100%, respectively [98,99]. IHC therefore, could be considered for use as a clinical screening mechanism for *CTNNB1* mutations in EC [98,99]. 

### 4.2. Tumorigenesis

Wnt/β-catenin signaling is involved in both the regulation of the normal endometrium and the aberrant development of endometrial hyperplasia or malignancy [16,95,100]. The normal endometrium undergoes cyclical, structural changes as part of the female menstrual cycle. During the proliferative phase, estrogen promotes proliferation of the endometrial glands and stroma, and during the luteal/secretory phase, progesterone induces endometrial differentiation and secretory activity. An imbalance in this cycle, particularly continuous unopposed estrogen, can result in endometrial hyperplasia and/or malignancy [95,100]. 

Wnt/β-catenin signaling is present in the endometrium and is regulated in the same cyclical fashion by estradiol (E2) and progesterone in both patient endometrium samples and EC cell lines [100]. Specifically, Wnt/β-catenin signaling is active during E2 exposure (proliferative phase). Progesterone exposure (secretory phase) inhibits Wnt/β-catenin signaling through induction of DKK1 and the transcription factor, FOXO1. Similarly, inhibition of progesterone with mifepristone (competitive antagonist) results in upregulation of Wnt/β-catenin signaling [101]. These findings are in line with the known function of Wnt/β-catenin signaling in stem cells: Wnt/β-catenin signaling activity (“Wnt-On”) promotes proliferation, while lack of Wnt/β-catenin signaling (“Wnt-Off”) allows for differentiation [100]. 

Correct regulation of Wnt/β-catenin signaling in this cyclical fashion is required for normal endometrial function [16,17,95,100], as loss of β-catenin results in squamous metaplasia of the endometrium, while constitutively active β-catenin results in endometrial hyperplasia [16,17]. The addition of unopposed estrogen to the models containing activating β-catenin mutations results in EC, demonstrating the relationship between Wnt/β-catenin signaling and hormonal regulation in the endometrium. This relationship is also highlighted by the fact that progesterone-mediated downregulation of Wnt/β-catenin signaling inhibits EC progression [17]. Progesterone’s downregulation of Wnt/β-catenin signaling may play a significant mechanistic role in its treatment effects. 

Other components of the Wnt/β-catenin signaling pathway have been implicated in the EC tumorigenesis. WNT7A is a Wnt/β-catenin signaling protein and is overexpressed in EC compared to normal endometrium and benign endometrial lesions. This overexpression is associated with worse clinical outcomes (shorter disease-free survival and overall survival) [46]. These findings again indicate that hyperactivation of Wnt/β-catenin signaling plays a role in the development and progression of EC. 

## 5. Cervical Cancer

Cervical cancer (CC) is the most common gynecologic malignancy in women worldwide and the third most common in the United States [51,102,103]. Human papilloma virus (HPV) 16 and HPV 18 are responsible for approximately 70% of CC cases, while other high-risk HPV (HR-HPV) types are responsible for approximately 20% of CC cases [103,104,105]. HPV proteins E6 and E7 promote tumorigenesis through inactivation of the tumor suppressors p53, Rb and p21. These tumor suppressors function by regulating the cell cycle and DNA repair pathways, therefore when inactivated by E6 and E7, cells undergo aberrant cell replication and accumulate DNA damage [104,105]. Although CC is primarily caused by HR-HPV, most HPV infections are cleared without causing cervical dysplasia, let alone cancer. Importantly, concomitant hyperactivation of Wnt/β-catenin signaling contributes to the progression of HPV-infection to tumor formation [18,104,105,106]. A variety of mutations and aberrations result in Wnt/β-catenin hyperactivation in CC [104,106,107,108]; based on TCGA, 83% of all CCs have at least one mutation within the Wnt signaling pathway [108] (Table 3). For instance, β-catenin transcriptional co-activators EP300 and CREBBP are mutated in 8% and 12% of cases, respectively. There is growing evidence that Wnt/β-catenin signaling plays a role in CC tumorigenesis and metastasis, and further research is needed. 

### 5.1. Tumorigenesis

Wnt/β-catenin signaling is hyperactivated in CC and plays an important role in HPV-dependent tumorigenesis [106]. WNT5A [19], as well as WNT4 and WNT8A, are overexpressed in HPV 16 positive CC [20]. WNT11 overexpression in CC is positively associated with HR-HPV E6 protein expression [21], and WNT11 and HR-HPV E6 expression are correlated with the progression of CC [22]. Interestingly, WNT7A is downregulated in CC cells, and restoration of WNT7A expression in CC cells results in decreased cell proliferation [109].

β-catenin is differentially expressed between CC and normal cervix, with multiple studies showing β-catenin expression in the nucleus and cytoplasm of CC cells compared to at the cell membrane in normal cervical tissue [110,111], suggesting enhanced β-catenin transcriptional activity in the former. HPV E6 and E7 proteins potentiate Wnt/β-catenin signaling by stabilizing β-catenin and by promoting β-catenin/TCF transcriptional activity [104,106,112,113,114]. In transgenic mouse studies, combined overexpression of E7 and β-catenin produced higher rates of transformation to CC compared to overexpression of either E7 or β-catenin alone [18]. 

In CC, there is crosstalk between Wnt/β-catenin and other oncogenic signaling pathways. Sal-like 4 (SALL4) is overexpressed in CC and promotes cell proliferation and tumor formation [115]. SALL4 increases levels of β-catenin and its target genes by directly binding to the *CTNNB1* promoter and trans-activating expression of *CTNNB1* [115]. Treatment with the TNKS inhibitor XAV-939 significantly decreases CC cell proliferation [115]. KIF18B is another identified oncogene associated with CC cell proliferation and invasion, and loss of KIF18B correlates with decreased cell proliferation and migration, as well as with a loss of β-catenin expression and its target genes [116]. Long noncoding RNA (lncRNA) CASC11 promotes cell proliferation and survival via Wnt/β-catenin signaling [117]. Treatment with DKK1, a negative regulator of the Wnt/β-catenin signaling pathway, leads to decreased cell survival [117]. DAX1 promotes cell growth, tumorigenicity, and tumorsphere formation through activation of the Wnt/β-catenin pathway [118] and transcriptionally represses *GSK3B*, preventing its expression and reducing the phosphorylation and proteasomal degradation of β-catenin [118]. Expression of SOX17 inhibits Wnt/β-catenin signaling activity in CC cells [119] and restrains the proliferation and tumor formation by transuppression of *CTNNB1* [119]. All of this taken together demonstrates the important role the Wnt/β-catenin pathway plays in the development and progression of CC. Suppression of Wnt signaling consistently attenuates cell growth; thus, it may serve as a targetable pathway to improve patient outcomes. 

### 5.2. Metastasis

CC is a disease that typically spreads by local extension. As such, the majority of CC cases that present with extra-cervical disease have local metastases to other pelvic organs. Distant metastases at the time of diagnosis are uncommon in CC (approximately 2%) and confer a poor prognosis with a 5-year survival rate of 16.5%. When distant metastases do occur, common sites include lung, bones, liver, and brain [103,120]. 

Wnt/β-catenin signaling has been found to play a role in the migration and invasion of CC. Both WNT5A and WNT11 promote CC cell proliferation and invasion, and both are also associated with CC metastasis and recurrence [19,22,121]. Sulfiredoxin (Srx) is an antioxidant enzyme that positively correlates with the progression of CC [23], and its expression is correlated with β-catenin expression [23]. Inhibition of the Wnt/β-catenin pathway with XAV-939 attenuates Srx expression and significantly inhibits invasion [23]. S100 calcium-binding protein A9 (S100A9) enhances proliferation and migration and induced EMT in CC [24], and β-catenin knockdown significantly suppresses this effect, suggesting that the effects of S100A9 are mediated through the Wnt/β-catenin signaling pathway [24]. HOTAIR is a lncRNA known to be associated with invasion and metastasis of several cancers, including cervical [25]. Knockdown of the HOTAIR inhibits the Wnt/β-catenin signaling pathway and EMT decreases cell proliferation and induces apoptosis in CC cells [25]. While this requires further investigation, these findings demonstrate that the Wnt/β-catenin signaling pathway plays an important role in metastasis of CC and may serve as a treatment target to improve outcomes for patients with metastatic CC. 

## 6. Targeting Wnt Signaling

**Figure 1:**
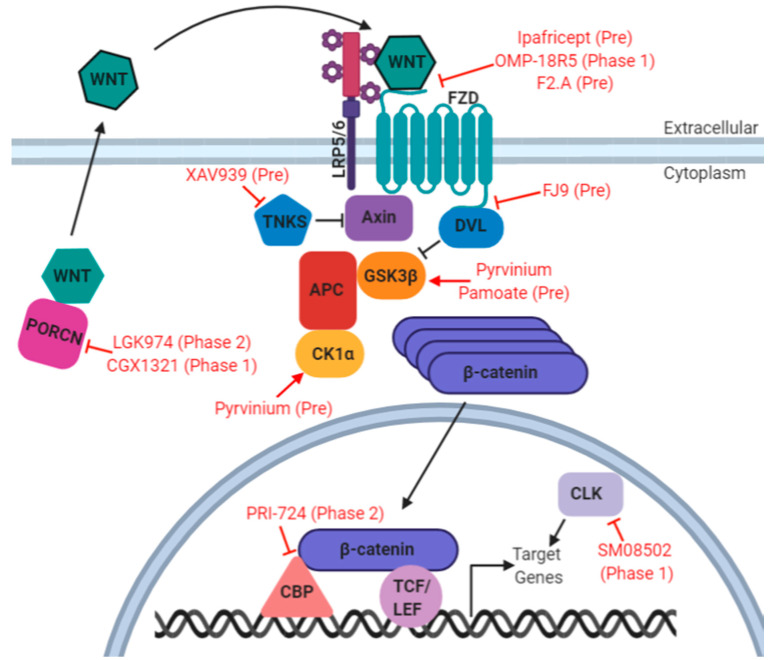
Therapeutic Targeting of the Wnt Signaling Pathway. Red letters = drug/compound name. Pre = pre-clinical development, Phase # = clinical trial phase. Clinical trial not necessarily for gynecologic malignancy. Figure generated with BioRender.

As described, Wnt/β-catenin signaling plays a pivotal role in tumorigenesis, metastasis, recurrence, and chemoresistance of EOC, EC, and CC, suggesting that it may serve as a targetable pathway in these cancers. Several inhibitors have been developed that different target nodes of this pathway (Figure 1).

### 6.1. PORCN Inhibitors

PORCN is essential for Wnt ligand secretion. Several inhibitors that target PORCN in the endoplasmic reticulum prevent the palmitoylation of Wnt proteins, which in turn prevents their secretion [3,35,122]. LGK974, or WNT974, is an orally available small molecule PORCN inhibitor that decreases EOC cell viability in vitro and blocks tumor growth in vivo [29,58,122]. In EOC mouse EOC models WNT974 decreases tumor growth and ascites formation and prolongs survival. These effects are enhanced when WNT974 is administered with paclitaxel [29]. There is currently a Phase 1 clinical trial investigating LGK974 as a single agent treatment for patients with solid malignancies for whom no effective standard treatment is available such as pancreatic cancer, triple-negative breast cancer, and cervical squamous cell carcinoma (NCT01351103) [29].

CGX1321, another PORCN inhibitor, inhibits both canonical and non-canonical Wnt signaling pathways [123]. A phase 1 clinical trial (NCT02675946) investigating single-dose escalation of CGX1321 in solid tumors should be completed in June 2020. In a syngeneic mouse model of EOC, CGX1321 treatment lead to increased overall survival, decreased tumor burden, and increased immune cell infiltration/function [28]. Inhibitors of Wnt production (IWPs) are also known to target PORCN as well as certain isoforms of CK1 such as CK1δ and CK1ε, possibly disrupting the β-catenin destruction complex [124]. While PORCN inhibitors continue to progress through clinical trials, recent discoveries of Wnt-secretion independent activation of Wnt signaling [125], suggest a possible mechanism of resistance that needs further investigation.

### 6.2. WNT/FZD Inhibitors

Wnt signaling may also be inhibited by direct binding to and inhibition of Wnt ligands and FZD receptors. Ipafricept (OMP54F28; IPA) is a recombinant fusion protein that competes with the FZD8 receptor and binds directly to Wnt ligands [30,126]. IPA was investigated in a phase 1b dose-escalation study in combination with paclitaxel and carboplatin in patients with recurrent platinum-sensitive ovarian cancer. The combination of IPA, paclitaxel, and carboplatin produced response rates and survival outcomes similar to historical treatment regimens; however, bone toxicities at efficacy doses prevented further testing of this treatment regime in EOC [30]. OMP-18R5 (vantictumab is a monoclonal antibody that inhibits cancer growth by targeting FZD1, FZD2, FZD5, FZD7, and FZD8 [127,128]. OMP-18R5 decreases tumor growth in xenografts of breast, pancreatic, colon, lung, and head and neck cancers [127,129] and is being evaluated in a number of phase I trials for these tumor types. However, it has not been studied in gynecologic malignancies. Pavlovic et al. utilized combinatorial antibody engineering to generate F2.A from OMP-18R5 to broaden the specificity to include FZD4 [128]. F2.A is specific to Wnt signaling and does not inhibit Norrin, which also signals through FZD4. FA.2 inhibits pancreatic cancer tumor growth in xenograft models [128], but this inhibitor has also not been tested in gynecologic malignancies. Carbamazapine, an antiepileptic drug, has recently been found to bind the cysteine-rich domain (CRD) of FZD8 [130]. Carbamazapine, an antiepileptic drug, has recently been found to bind the cysteine-rich domain (CRD) of FZD8 [130], suggesting that carbamazapine may be worth exploration as a treatment in gynecologic malignancies.

### 6.3. DVL Inhibitors

DVL is important for transducing Wnt signals by recruiting components of the destruction complex to the cell membrane [36,131]. In order for DVL to function, it binds to the cytoplasmic tail of FZD proteins through its PDZ domain [132]. FJ9 is an inhibitor that disrupts the interaction between FZD and the PDZ domain of DVL. FJ9 was confirmed to downregulate Wnt/β-catenin signaling and suppress tumor cell growth in cervical, lung and colorectal cancer lines in vitro, as well as in a lung cancer xenograft [133]. 

### 6.4. Destruction Complex Inhibitors

Stabilizing the β-catenin destruction complex can lead to increased ubiquitination of β-catenin, making it an attractive drug target. Pyrvinium, an FDA approved drug, binds all CK1 family members in vitro, selectively potentiating CK1α kinase activity [134]. Colon cancer cells with APC mutations were sensitive to pyrvinium treatment with a decrease in both Wnt signaling and cell proliferation, suggesting that cancers with mutations in the Wnt/β-catenin pathway such as EC may be sensitive to pyrvinium as well [134]. Indeed, pyrvinium inhibits platinum-resistant EOC tumor growth and induces apoptosis in vitro and *in vivo*, and these effects are enhanced when pyrvinium is combined with paclitaxel. Pyrvinium has these effects on EOC by decreasing β-catenin levels and suppressing β-catenin-mediated transcription; when β-catenin is stabilized or overexpressed, EOC cells are no longer impacted by pyrvinium [31]. 

Pyrvinium pamoate (Pyr. Pam.) is also an FDA approved drug that stabilizes GSK-3β potentially through inhibition of Akt/PI3K, leading to decreased levels of β-catenin and its downstream targets [135]. Pyr. Pam. inhibits proliferation and invasion of endometrial stromal cells from endometriosis specimens [136] but has not yet been studied in EC cells. Our group has previously demonstrated that treatment with Pyr. Pam. is sufficient to sensitize ovarian cancer cells to PARPi and leads to decreased tumor size and ascites volume in vivo [26].

TNKS proteins are PARPs that can regulate the destruction complex [137]. TNKS poly-ADP ribosylates (PAR) Axin, which in the absence of Wnt/β-catenin signaling, leads to proteasomal degradation and in the presence of Wnt/β-catenin signaling can stabilize the interaction between Axin and LRP5/6 [137]. XAV939 is a TNKS inhibitor that leads to decreased β-catenin-dependent transcription through its regulation of Axin [122,138]. XAV939 treatment decreases the viability of EOC cell lines and increases radiosensitivity in CC cells [27,32]. 

### 6.5. Transcriptional Co-Activators/Target Gene Inhibitors

There are a number of co-activators of β-catenin-dependent transcription, including CREB binding protein (CBP) [35]. In Phase 1 clinical for patients with hepatitis C virus-related cirrhosis, intravenous PRI-724 (which inhibits the interaction between CBP and β-catenin [139]) over 12 weeks was well tolerated [140]. In chemotherapy-resistant EOC with hyperactivated β-catenin/CBP signaling, PRI-724 was able to induce sensitization to platinum therapy [33]. 

Several small molecules have been designed that inhibit CDC-like kinase (CLK) activity [141], thereby inhibiting Wnt/β-catenin gene expression through alternative splicing of transcribed RNA [141]. SM08502 reduces Wnt/β-catenin signaling and can be orally administered to significantly inhibit the growth of GI tumors in xenograft mouse models [141]. SM08502 is currently being investigated in Phase 1 clinical trial for patients with advanced solid tumors (NCT03355066).

### 6.6. Wnt Inhibitor Toxicities

As Wnt signaling is highly conserved and complex, playing an important role in many biological processes, targeting the Wnt signaling pathways carries a risk for significant side effects and toxicities [142,143]. The role of Wnt signaling in tissue homeostasis seems to be a particular source of toxicity, specifically in bone, intestinal, skin, and hair homeostasis, as well as in hematopoiesis [142]. As previously described, a phase Ib study evaluating IPA in combination with paclitaxel and carboplatin in patients with recurrent platinum-sensitive ovarian cancer demonstrated clinical activity but was limited by the toxicity of fragility fractures [30]. Evaluation of Wnt inhibitors in other disease types has demonstrated toxicities, including loss of bone density, liver injury, enteritis, and thrombocytopenia [122,140,144,145]. Further development and evaluation of Wnt inhibitors are needed to find ways to more precisely and safely target Wnt signaling in gynecologic malignancies. 

## 7. Conclusions

Wnt signaling impacts EOC, EC, and CC in various ways, differing between cancer types and disease phases. Although the exact mechanisms are not yet clear, it is evident that Wnt/β-catenin signaling plays a vital role in EOC’s therapy resistance, recurrence in EC, metastasis in EOC and CC, and tumorigenesis for all three cancer types. Studies that have demonstrated Wnt/β-catenin signaling activity in gynecologic malignancies have not only illuminated important behaviors of these cancers but have also shed light on possible targets in the Wnt/β-catenin signaling pathway for future treatments. 

Targeted therapies against Wnt/β-catenin signaling are beginning to be evaluated in various cancer types, but further research evaluating Wnt/β-catenin signaling inhibitors in gynecologic malignancies is needed. Looking toward the future of inhibiting Wnt signaling in gynecologic malignancies and reducing systemic toxicities, research is needed for improved targeting of drugs to specific tissue. For instance, utilizing antibody–drug conjugates that target specific FZD receptors could increase cancer cell specificity and inhibit tumor progression. Further, given the contribution of Wnt signaling to immune cell response, there is strong rational to evaluate combinatorial immunotherapies and Wnt inhibitors. 

## Figures and Tables

**Table 1:** Canonical and non-canonical Wnt/β-catenin signaling pathway alterations in high grade serous ovarian cancer. The Cancer Genome Atlas (TCGA), Firehose Legacy. Wnt/β-catenin KEGG Pathway (150 genes). AMP = amplification, HOMDEL = homozygous deletion, Mut = mutated.

TCGA, OVCA, Firehose Legacy					
Wnt/Beta-Catenin Pathway	Gene	AMP	HOMDEL	Mut	Altered (AMP+HOMDEL+Mut)
Degradation Complex	APC	1.38%	2.94%	2.22%	6.54%
Degradation Complex	CSNK2A1	8.29%	0.17%	0.32%	8.78%
Degradation Complex	CSNK2B	6.39%	0.00%	0.32%	6.71%
Inhibitor	DKK4	5.87%	0.17%	0.00%	6.04%
Ligand	CER1	3.97%	1.38%	0.00%	5.35%
Ligand	WNT11	9.84%	0.17%	0.63%	10.65%
Ligand	WNT16	7.08%	0.52%	0.95%	8.55%
Ligand	WNT2	6.91%	0.35%	0.32%	7.57%
Ligand	WNT3A	7.94%	0.00%	0.00%	7.94%
Ligand	WNT5B	11.92%	0.00%	0.00%	11.92%
Ligand	WNT7B	0.35%	6.56%	0.63%	7.54%
Ligand	WNT9A	7.77%	0.00%	0.63%	8.41%
Receptor	FZD3	0.86%	6.04%	0.00%	6.91%
Receptor	FZD4	10.02%	0.17%	0.00%	10.19%
Receptor	FZD6	20.90%	0.17%	0.00%	21.07%
Receptor	LRP5	5.35%	0.17%	0.95%	6.48%
Receptor	LRP6	10.02%	0.17%	0.32%	10.51%
Signaling	CACYBP	5.87%	0.00%	0.00%	5.87%
Signaling	DAAM2	5.35%	0.00%	0.63%	5.99%
Signaling	DVL1	3.97%	1.38%	0.00%	5.35%
Signaling	DVL3	26.77%	0.00%	0.32%	27.09%
Signaling	PLCB1	7.08%	0.17%	1.27%	8.52%
Signaling	PLCB4	6.04%	0.00%	0.32%	6.36%
Signaling	PPARD	6.04%	0.00%	0.63%	6.68%
Signaling	PPP2CB	1.21%	4.32%	0.00%	5.53%
Signaling	PPP2R5A	4.32%	0.52%	0.32%	5.15%
Signaling	PPP2R5D	5.70%	0.17%	0.32%	6.19%
Signaling	PPP3CC	0.17%	7.25%	0.32%	7.74%
Signaling	PRKACA	15.37%	0.00%	0.00%	15.37%
Signaling	VANGL2	5.01%	0.00%	0.32%	5.33%
Secretion	PORCN	6.56%	0.69%	0.63%	7.89%
Transcriptional Target/Regulation	CCND1	6.74%	0.00%	0.00%	6.74%
Transcriptional Target/Regulation	CCND2	11.57%	0.17%	0.00%	11.74%
Transcriptional Target/Regulation	CCND3	6.04%	0.17%	0.00%	6.22%
Transcriptional Target/Regulation	CHD8	4.15%	0.35%	1.59%	6.08%
Transcriptional Target/Regulation	CREBBP	1.55%	2.59%	2.22%	6.37%
Transcriptional Target/Regulation	CTBP1	6.91%	0.69%	0.32%	7.92%
Transcriptional Target/Regulation	CTBP2	5.87%	1.04%	0.32%	7.23%
Transcriptional Target/Regulation	CUL1	11.23%	0.69%	0.00%	11.92%
Transcriptional Target/Regulation	JUN	4.66%	0.86%	0.00%	5.53%
Transcriptional Target/Regulation	MMP7	7.60%	0.52%	0.32%	8.43%
Transcriptional Target/Regulation	MYC	41.97%	0.00%	0.00%	41.97%
Transcriptional Target/Regulation	NFATC2	8.46%	0.17%	0.32%	8.95%
Transcriptional Target/Regulation	NKD2	14.34%	0.17%	0.00%	14.51%
Transcriptional Target/Regulation	RAC3	6.91%	0.86%	0.00%	7.77%
Transcriptional Target/Regulation	RUVBL1	5.53%	0.00%	0.00%	5.53%
Transcriptional Target/Regulation	SENP2	26.60%	0.00%	0.00%	26.60%
Transcriptional Target/Regulation	SOX17	11.23%	0.00%	0.00%	11.23%
Transcriptional Target/Regulation	TBL1XR1	28.67%	0.00%	0.32%	28.99%
Transcriptional Target/Regulation	TP53	1.38%	0.35%	87.62%	89.35%

100 genes with less than 5% altered.

**Table 2:** Canonical and non-canonical Wnt/β-catenin signaling pathway alterations in Uterine Corpus Endometrial Carcinoma. The Cancer Genome Atlas (TCGA), Firehose Legacy. Wnt/β-catenin KEGG Pathway (150 genes). AMP = amplification, HOMDEL = homozygous deletion, Mut = mutated.

TCGA, EC, Firehose Legacy					
Wnt/Beta-Catenin Pathway	Gene	AMP	HOMDEL	Mut	Altered (AMP+HOMDEL+Mut)
Degradation Complex	APC	0.41%	0.00%	11.98%	12.40%
Receptor	LRP6	0.83%	0.00%	7.85%	8.68%
Signaling	CTNNB1	0.00%	0.41%	29.75%	30.17%
Signaling	DVL3	7.02%	0.83%	3.72%	11.57%
Signaling	PPP2R1A	0.83%	0.41%	10.74%	11.98%
Signaling	PRKACA	5.79%	0.00%	2.48%	8.26%
Signaling	ROCK2	2.89%	0.00%	5.79%	8.68%
Transcriptional Target/Regulation	CCND1	3.31%	0.00%	6.20%	9.50%
Transcriptional Target/Regulation	CHD8	0.41%	0.00%	7.85%	8.26%
Transcriptional Target/Regulation	CREBBP	1.65%	0.41%	9.09%	11.16%
Transcriptional Target/Regulation	EP300	1.65%	0.00%	9.09%	10.74%
Transcriptional Target/Regulation	MYC	7.02%	0.00%	3.31%	10.33%
Transcriptional Target/Regulation	SENP2	6.20%	0.83%	2.89%	9.92%
Transcriptional Target/Regulation	SOX17	6.61%	0.00%	2.89%	9.50%
Transcriptional Target/Regulation	TBL1XR1	6.61%	0.41%	4.96%	11.98%
Transcriptional Target/Regulation	TP53	0.00%	0.00%	28.10%	28.10%

126 genes with less than 5% altered.

**Table 3:** Canonical and non-canonical Wnt/β-catenin signaling pathway alterations in Cervical Squamous Cell Carcinoma and Endocervical Adenocarcinoma. The Cancer Genome Atlas (TCGA), Firehose Legacy. Wnt/β-catenin KEGG Pathway (150 genes). AMP = amplification, HOMDEL = homozygous deletion, Mut = mutated.

TCGA, CC, Firehose Legacy					
Wnt/Beta-Catenin Pathway	Gene	AMP	HOMDEL	Mut	Altered (AMP+HOMDEL+Mut)
Receptor	FZD6	6.89%	0.13%	0.39%	7.41%
Signaling	DVL3	17.73%	0.00%	0.20%	17.93%
Signaling	PRKACA	7.65%	0.38%	0.39%	8.42%
Transcriptional Target/Regulation	MMP7	5.99%	0.51%	0.39%	6.89%
Transcriptional Target/Regulation	MYC	21.43%	0.00%	0.20%	21.63%
Transcriptional Target/Regulation	NKD2	7.53%	0.26%	0.20%	7.99%
Transcriptional Target/Regulation	SENP2	17.35%	0.00%	0.20%	17.55%
Transcriptional Target/Regulation	TBL1XR1	19.26%	0.38%	0.98%	20.62%
Transcriptional Target/Regulation	TP53	0.38%	0.51%	61.18%	62.07%

135 genes with less than 5% altered.
